# Investigating the accuracy of adjusting for examiner differences in multi-centre Objective Structured Clinical Exams (OSCEs). A simulation study of video-based Examiner Score Comparison and Adjustment (VESCA)

**DOI:** 10.1186/s12909-024-06462-3

**Published:** 2024-12-18

**Authors:** Peter Yeates, Gareth McCray

**Affiliations:** School of Medicine, Keele University, David Weatherall Building, Keele, ST5 5BG UK

**Keywords:** Equivalence, Simulation, Many Facet Rasch Modelling

## Abstract

**Introduction:**

Ensuring examiner equivalence across distributed assessment locations is a priority within distributed Objective Structured Clinical Exams (OSCEs) but is challenging as examiners are typically fully nested within locations (i.e. no overlap in performances seen by different groups of examiners). Video-based Examiner Score Comparison and Adjustment (VESCA) is a recently developed method which uses video-based linking to compare and (potentially) adjust for the effect of different groups of examiners within OSCEs. Whilst initial research on VESCA has been promising, the accuracy of the resulting adjusted scores is unknown. Given this, we aimed to investigate the accuracy of adjusted scores produced by VESCA under a range of plausible operational parameters.

**Methods:**

Using statistical simulation, we investigated how: 1/proportion of participating examiners, 2/ number of linking videos, 3/baseline differences in examiner stringency between schools (i.e. whether examiners in School A are, on average, more stringent than the examiners in School B), 4/number of OSCE stations and 5/different degrees of random error within examiners’ judgements influenced accuracy of adjusted scores.

We generated distributions of students’ “true” performances across several stations, added examiner error, and simulated linking through crossed video-scoring (as occurs in VESCA). We then used Many Facet Rasch Modelling to produce an adjusted score for each student which we compared with their corresponding original “true” performance score. We replicated this 1000 times for each permutation to determine average error reduction and the proportion of students whose scores became more accurate. Simulation parameters were derived from a real, summative, whole curriculum undergraduate Year 3 OSCE at Keele University School of Medicine.

**Results:**

We found that in all conditions where no baseline difference existed between groups of examiners, score adjustment only minimally improved or even worsened score accuracy. Conversely, as the size of baseline differences between schools increased, adjustment accuracy increased, reducing error by up to 71% and making scores more accurate for up to 93% of students in the 20% baseline-difference condition.

**Conclusions:**

Score adjustment through VESCA has the potential to substantially enhance equivalence for candidates in distributed OSCEs in some circumstances, whilst making scores less accurate in others. These findings will support judgements about when score adjustment may beneficially aid OSCE equivalence.

**Supplementary Information:**

The online version contains supplementary material available at 10.1186/s12909-024-06462-3.

## Introduction

Rater-based assessments are well known to suffer from a range of construct-irrelevant influences, such as rater stringency, range restriction and bias [1]. Within simulated performance testing in medical education (such as objective structured clinical exams (OSCSs)), a further problem occurs: owing to student numbers, assessments are typically distributed across multiple parallel tracks of (ostensibly) the same exam, or indeed across widely spaced geographical locations. This raises the potential that examiners in different locations may hold systematically different frames-of-reference when judging performance [2], for example if local practice norms, resources [3] or conceptions of competence [4] vary. Whilst a few studies have illustrated this potential [5, 6] it is rarely studied as assessment designs are typically fully nested meaning these effects are confounded with student ability making them challenging to explore. Inter-site examiner variability (when present) can be considered a key threat to the “scoring” domain of Kane’s validity model [7] as it adds construct-irrelevant variance to scores. As scoring is the first step in the inferential chain of this validity model, it would be expected to influence all subsequent inferences and so is critical to all further interpretation of assessment scores.

Examiner variability in rater-based assessments has typically been addressed through a mixture of assessment deign [8], examiner training [9] and consideration of different rating formats, however neither reformulating marksheets [10–12] nor rater training [13, 14] have achieved large improvements in examiner variability. Psychometric monitoring of assessments can be performed with simple estimates of reliability (e.g. Cronbach’s alpha [15]) or generalizability theory [16], however neither of these techniques is suited to detecting inter-site differences in fully nested OSCE designs for the reasons already described.

Recently Yeates et al. [17] have developed a method called video-based examiner score comparison and adjustment (VESCA) which uses video-based linking to overcome this challenge, but the accuracy of the score adjustments it makes are unknown. VESCA employs three sequential phases: 1/ a sample of candidates are videoed on each of the tasks (known as “stations”) within the OSCE; 2/ all examiners, in addition to judging live candidates, are asked to score a small number of videos of student performances from the station they examined; 3/ the partial crossing created by the video-scores is used to link different examiner groups (“examiner-cohorts”) within statistical analyses to compare and equate for examiner effects. Notably, therefore, whilst examiners from different locations all score the same videos of student performances, the scores they allocate to videos do not directly contribute to the scores for those students but are instead used to model examiner differences which can (if desired) be used to calculate adjusted scores for students based on the measured examiner differences. Yeates et al. have used VESCA within a number of studies, in each case showing differences between the estimates of different examiner-cohorts ranging from 5.7% [17], 6.9% [18] to 7.1% [19]. Resulting score adjustments suggested that a proportion of students would vary their pass/fail classification (up to 16% depending on cut score [19]) or their rank position [18]. Critically, as the authors acknowledge in each paper, these observations depend on a strong assumption that the adjusted scores produced by VESCA (generally through Many Facet Rasch Measurement) are indeed more accurate representations of candidates’ true performance than their raw scores. Yeates et al. [20] used subset re-sampling from Yeates et al.’s 2021 data to explore this potential. By varying the number of linking videos per participating examiner and the proportion of examiners who scored videos, they showed that candidates’ score adjustments (i.e. the difference between their adjusted and raw scores) were sensitive to changes in both of these parameters. The purpose of this current study is to extend that work, by determining the accuracy of the adjusted scores produced by VESCA, and to explore how that accuracy varies under a plausible range of different operational parameters.

Several parameters could conceivably influence the accuracy of score estimates produced by VESCA. As Yeates and McCray [20] have previously shown that firstly examiner participation rates and secondly the number of linking videos scored by each examiner can both influence score adjustments, these variables seem germane to understanding VESCA’s accuracy. Theoretically, we would expect that with greater amounts of linking (i.e. more videos per station; greater examiner participation), the Many Facet Rasch model would develop more accurate estimates of examiner-cohort effects as the impact of random variability on these estimates would be reduced. Third, OSCEs frequently vary in their number of constituent stations, which has a significant influence on reliability [21]. Consequently, station numbers could influence VESCA’s accuracy. Fourth, Many Facet Rasch Modelling can adjust for systematic variations between examiners, but prior research has shown that a significant proportion of score variance in OSCEs is random or unexplained [22]. As a result, determining the impact of different levels of random variability on VESCA’s score adjustments is important.

Lastly the express purpose of VESCA is to compare examiners’ influence across distributed sites where examiners and students are nested together (i.e. no crossover between the candidates seen by different groups of examiners). Prior research has suggested that inter-site variations may account for up to 16–17% of score variance in some instances [5, 6]. Further work has suggested that examiners frame of reference relates to the typical standard of performance to which they are exposed [23]. Consequently, it is conceivable that location A could have highly capable candidates and stringent examiners, whereas location B could have less capable candidates and lenient examiners. Notably, whilst examiner equivalence would be highly different between these locations, unadjusted OSCE scores could be very similar. We refer to this potential systematic difference in examiner stringency between different institutions effect as “examiner baseline differences”. As its ability to adjust for these effects is critical to the intended use of VESCA, we additionally sought to understand the influence of baseline differences on the accuracy of VESCA score adjustments.

To operationalise “accuracy” in practical terms we considered 1/ the proportion of candidates whose scores became more accurate and 2/ the reduction in total error variance in each scenario. We then asked the following research questions:

1. How is the accuracy of score estimates produced by VESCA influenced by:
  - The number of linking videos per examiner (0, 2,4,6, or 8 linking videos)
  - The proportion of examiners who participate in scoring videos (50%, 65%, 80%, 100%)
  - The combination of these 2 effects
2. How is the accuracy of score estimates produced by VESCA influenced by:
  - Differing extents of baseline differences in examiner stringency between different sites (0%, 5%, 10%, 20%)
  - The number of stations in the OSCE (6, 12, or 18 stations)
  - The combination of these two effects
3. How is the accuracy of score estimates produced by VESCA influenced by reduction in the degree of random variability in examiners’ scoring (random error divided by 2, by 4, and by 8)

We considered one further issue as an ancillary research question. Given that some degree of imprecision is inevitable in all statistical modelling, it seemed plausible that the accuracy of adjusted scores may relate to the size of the adjustment being made, i.e. large adjustments may be more accurate than small adjustments, because the ratio of size of the adjustment to the size of the imprecision may be greater (i.e. a greater signal to noise ratio). This may enable a score adjustment threshold to be determined above which score adjustments reach greater accuracy. We examined this within all data produced by studies 1–2, by asking:

4. How does the proportion of candidates whose scores become more accurate vary for different sizes of score adjustment for each of the parameters investigated within RQs 1–2.

## Methodology

### Simulated data generation

**Fig. 1:**
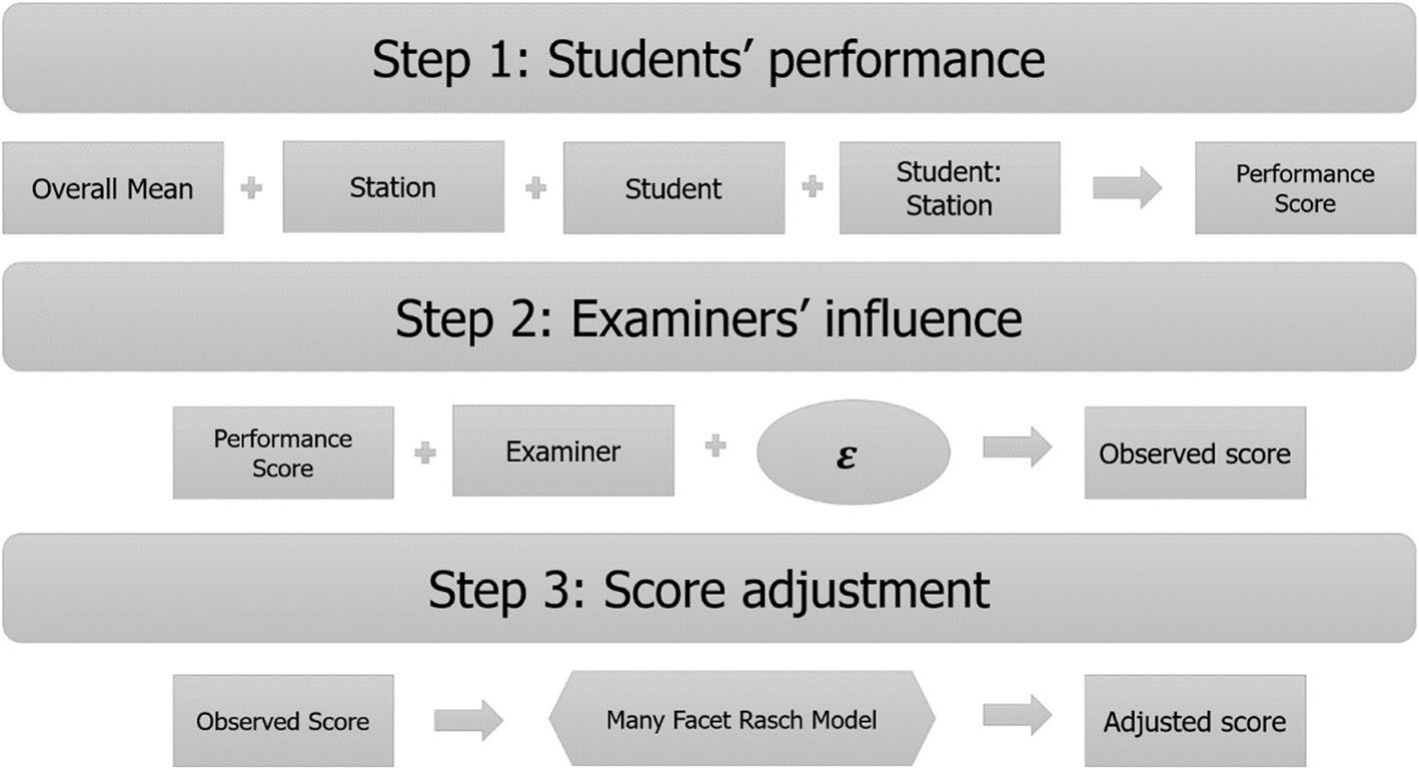
A conceptual diagram of the data simulation process

We simulated the operation of VESCA through three sequential processes (see Fig. 1), by simulating the combined effect of several known influences on OSCE scores. All parameter estimates were empirically-derived from analysis of prior data from a study by Yeates et al.’s [19] in which VESCA had been used in a real OSCE. The OSCE in Yeates et al.’s study was a 12 station, in-person, summative undergraduate OSCE for year 3 (out of 5) students (*n* = 113) who were in their first year of predominantly clinically-based learning at Keele University school of Medicine. The OSCE sampled the whole curricular content of that year of study, involving a broad-based range of case presentations from medicine, surgery, general practice, psychiatry and child health. Stations integrated information gathering, clinical reasoning, physical examination skills, procedural skills, patient management and communication skills and were scored via the GeCoS domain-based rating scale [24, 25]. Participating examiners additionally scored 4 videos of performances of students on the station which they examined (but not necessarily students they had encountered in the live exam) to produce partial crossing in the data. Video score data comprised 17.7% of the total data. We derived estimates of range of student performance from the overall data in Yeates et al.’s study and estimates of examiner variability from the crossed video score data.

Firstly, we modelled the “true” performance of a range of students on each station in an OSCE using a simple sum-score approach. Data were generated using the GeCos scale [24] which combines ratings on several performance domains to give a scale minimum of 6 and maximum of 27. To do this we randomly generated a distribution of students’ overall ability (M = 19.47 out of 27; SD = 1.13 (5.4% of scale)) and then generated a range of station difficulties (SD 1.52 (7.2% of scale)) and an idiosyncratic student_x_station interaction (SD = 1.71 (8.1% of scale)). We combined these, using a linear function to produce students’ simulated “true” performance on each station in the OSCE.

Secondly, we added examiner variability to these scores by creating a distribution of examiners (SD = 1.40 (6.7% of scale)). Examiners were randomly allocated to a station and to 1 of 4 examiner cohorts (i.e. distinct groups of examiners) such that each students’ “true” scores were exposed to a unique group of examiners stringencies, and the same examiner stringency applied to all students for a given station within a cohort. As examiners did not change station, we could not model examiner_x_station effects. Next, we simulated an additional random error term (SD = 2.35 (11% of scale)) to capture additional unmodeled variation in examiners’ scoring (for example due to the time of day [26], contrast [27] or halo [28] effects from the previous candidates, examiner_x_student interactions, and any other unknown sources of variability). We summed the students’ “true” performance score on each station, with the examiner stringency and the additional random term to give the student’s “observed score” on each station in the OSCE – the scores they would have actually received in the exam. Formally, generation of the observed students’ scores can be expressed as:

$$\:{Score}_{ijk}=\:{\beta\:}_{0}+{u}_{1}{Station}_{i}{\:+\:u}_{2}{Student}_{k}{\:+\:u}_{3}{Student:Station}_{ik}+{\:u}_{4}{Examiner}_{j}+\:{\epsilon\:}_{ijk}$$

Where: $\:{\beta\:}_{0}$ the overall model intercept (i.e., average student score in the dataset), $\:{u}_{1}$ station difficulty $\:i$, $\:{u}_{2}$ student ability $\:k$, $\:{u}_{3}$ the interaction between student $\:k$ and station$\:\:i{,\:u}_{4}$ examiner $\:j$ stringency, and $\:{\epsilon\:}_{ijk}$ is the residual error.

Thirdly, we mimicked the influence of the VESCA procedure by randomly selecting a specified number of student performances on each station and nominating these as “video performances”. A proportion of examiners were then randomly selected to “participate” (see RQ 3) and the stringency values of these examiners + the random error term were applied to the relevant “video performances” for the station they had examined. This created an additional set of crossed “video scores” for each station as would be collected by using VESCA (i.e. the same “video performances” were scored by multiple examiners from different examiner cohorts). This created a dataset comprised of students’ “live” observed scores on each station in the OSCE, and further observed video scores allocated to station-specific videos by examiners. All data generation was performed via a flexible function written in R [29]. The function always has four cohorts of examinees but allows the manipulation of i) the number of linking videos, ii) the min and max of the score range, iii) the numbers of stations, iv) the number of candidates, v) the number of cohorts, vi) the number of examiners, vii) the mean ability of a candidate, viii) the standard deviation of candidate scores, ix) the standard deviation of station difficulties, x) the standard deviation of examiner stringencies, xi) the standard deviation of a station by candidate interaction (i.e., the error in the ‘performance score’) and xii) the expected proportion of examiners who would participate in the linking process. See Fig. 1 for details.

### The Many-Facet Rasch model

As in the procedures used by Yeates et al. [19], these data were then analysed using Many Facet Rasch Modelling, in FACETS [30] to produce an adjusted overall (i.e. average) score for each student (see Fig. 1). The Many-Facet Rasch Model (MFRM) [31] expands the simple two parameter Rasch model [32], which focuses on item difficulty and student ability, to include additional facets to model effects such as rater leniency, schools, locations, etc. A simple, three facet model could be expressed as:

$$\:log\left(\frac{{P}_{nijk}}{{P}_{nij(k-1)}}\right)={B}_{n}-{D}_{i}-{C}_{j}-{F}_{k}$$

Where, $\:{P}_{nijk}$ is the probability that person *n*, on item *i* by judge *j*, is given a rating of *k*. $\:{P}_{nij(k-1)}$ is the probability that person *n*, on item *i* by judge *j*, is given a rating of *k-1*, $\:{B}_{n}$ is the ability measure of the test taker *n*, $\:{D}_{i}$ is the ‘difficulty’ of test item *i*, $\:{C}_{j}$ is the severity of rater *j*, and $\:{F}_{k}\:$relates to the probability of being assessed in category *k* of item *I*, rather than category *k-1*. Applying this within our study, the specific model used was:

$$\:log\left(\frac{{P}_{nijk}}{{P}_{nij(k-1)}}\right)={Student}_{n}-{Station}_{i}-{Cohort}_{j}-{F}_{k}$$

Which models the probability of student *n* responding to station *i*, examined by an examiner in examiner cohort *j* being rater in category *k* on item *i*, rather than category *k-1*.

We ran each simulation 1000 times in order to obtain stable estimates. As this was computationally demanding, simulations were run via 16 virtual machines on a 16-core server each linking R to facets using the R package “immer” [33].

### Simulations

Several simulations were conducted to mimicking the VESCA method in various contexts. Unless otherwise specified, simulations modelled 12 stations, 60 students in 4 cohorts with 48 examiners, with an assumed 80% of examiners participating, and 4 linking videos.

#### Study 1

The first study addressed RQ1 by modifying the number of linking videos (0, 2, 4, 6 and 8) and the expected proportion of examiners to consent to providing linking data (50%, 65%, 80% and 100%). This included modelling “typical” conditions (i.e. Yeates et al. 2021 [19]) which comprised 4 linking videos and 80% participating examiners. No baseline differences between schools were modelled in study 1. All permutations of parameter values were simulated for a total of 5 (range of linking videos) x 4 (range of examiner participation rates) = 20 sets of 1000 simulations for each unique pair of values.

#### Study 2

The second study addressed RQ2, by looking at the effect of changing the number of stations [6, 12, 18] and the degree of site-related baseline difference in examiner stringency / student leniency (0%, 5%, 10%, 20%) – see last paragraph of background for definition. Baseline differences were modelled selecting 2 examiners-cohorts as “school A” and 2 examiner cohorts as “school B” and then adding or subtracting the relevant percentage score to the students and examiners coefficients for each school. We assumed that examiner stringency was completely negatively correlated with student ability (i.e., as students became more able, examiners were more stringent and thus the mean expected scores between sites would be equal). All possible combinations of parameter values were simulated for a total of 3 (numbers of stations) x 4 (degrees of baseline difference) = 12 sets of 1000 simulations for each unique pair of values.

#### Study 3

The third study examined RQ3 by reducing the size of the overall residual error term on the performance of the VESCA linking model. This was done by dividing the error term by 2 (error/2 – i.e. 50% of error in prior studies); by 4 (error/4, 25% of the error in prior studies) or by 8 (error/8, 12.5% of the error in prior studies). The objective of this study was not to investigate a plausible real-life situation (as reducing the residual error is a very difficult to achieve) but to understand the impact that this residual score error was having on the functioning of VESCA.

### Measurement of performance

Having generated data using these parameters and subsequently obtained FACETS estimates of each students’ adjusted score, we used them to determine accuracy of the estimates.

To do this, we calculated three variables for each student, for all 1000 iterations of each permutation of each study:

A. Observed Score Error: The mean absolute difference (MAD) of the observed score – the performance score. This gave the residual error of each student’s observed score, from their “true” score, prior to adjustment.
B. Adjusted Score Error: The mean absolute difference (MAD) of the adjusted score – the performance score. This gave the residual error of each student’s score, from their “true” score, after adjustment via the VESCA method.
  For the VESCA method to show utility, we would expect the adjusted scores to be closer to the “true” scores than the observed scores. Lastly, we calculated:
C. Score Adjustment: The mean absolute difference of the adjusted score – the observed score.

This gave the size of the adjustment made to each student’s score using the VESCA method.

We then calculated the first of our dependent variables: the proportion of students whose adjusted score became more accurate than their observed score (for brevity, termed “pAcc”). This was defined as the proportion of students for whom “adjusted score error” < “observed score error” (i.e. VESCA score adjustment had resulted in a score nearer to their “true” performance score).

For each permutation of each study, we then calculated:

1. The mean of all students “observed score error”
2. The mean of all students “adjusted score error”
3. The ratio of mean “adjusted score error”: mean “observed score error” (i.e. 1. / 2.)

This demonstrated, on average, how much score accuracy changed for each permutation in each study. For brevity, we term this the “error ratio” (ErR), noting that values below 1 indicated improved accuracy and values above 1 indicated reduced accuracy.

To address RQ4 (how does the proportion of candidates whose scores become more accurate vary for different sizes of score adjustment), we categorised each students’ data in each permutation of each study, based on the size of the score adjustment they received, using categories of score adjustment (expressed as a percentage of the assessment scale) of: [0–1%), [1–2%), [2–3%), [3–4%), [4–5%), [5–6%), [6–7%), [7–8%), [8–9%), (> 9%). Next, we further categorised students based on the extent of change in the accuracy of their adjusted scores compared to their observed scores (i.e. how much more or less accurate their adjusted score became), using categories also based on percent of the assessment scale of (<−6%), (−6%—4%], (−4%—−2%], (−2%—0%], [0–2%), [2—4%), [4—6%), (> 6%). We then tabulated these results for inspection. To aid categorisation of these findings, we used a target of 80% of students’ scores becoming more accurate in order to define whether a useful threshold could be established.

## Results

Data were generated in 35 separate simulations, resulting in 25,200,000 “performance” scores (i.e. scores for 2,100,000 students on an average of 12 stations).

### Research question 1

How is the accuracy of VESCA score estimates influenced by:

- The number of linking videos per examiner (0, 2,4,6, or 8 linking videos)
- The proportion of examiners who participate in scoring videos (50%, 65%, 80%, 100%)
- The combination of these 2 effects

These questions were addressed by study 1. The accuracy of adjusted scores across all parameters modelled in this study were low. Notably, this study assumed that there were no baseline differences between examiners in different sites. Error ratio (ErR) values ranged from a worst case 1.22 (i.e. adjusted scores contained 22% *more* error than observed scores) for 2 linking videos, with 50% examiner participation, to a best case of 0.94 (i.e. score adjustment removed 6% of the error in the observed scores) for 8 linking videos with 100% examiner participation. The proportion of students whose scores became more accurate (pAcc) as a result of adjustment corresponded closely, ranging from pAcc = 0.44 (44% of students’ scores became more accurate; 56% of students’ scores became *less* accurate) for 2 linking videos / 50% examiner participation, to pAcc = 0.53 (53% of students’ scores became more accurate) for 8 linking videos / 100% examiner participation. A detailed breakdown of all permutations of these parameters can be seen in Table 1.

**Table 1:** Influence of number of linking videos per examiner and proportion of participating examiners on adjusted score accuracy

Number of Linking Videos per Examiner	Proportion of participating examiners	Mean Error in Observed scores (SD)	Mean Error in Adjusted Scores (SD)	Error ratio	Proportion of students' whose scores became more accurate through adjustment
0	50	0.603 (0.46)	0.623 (0.47)	1.03	0.48
0	65	0.605 (0.46)	0.619 (0.47)	1.02	0.48
0	80	0.605 (0.46)	0.618 (0.47)	1.02	0.49
0	100	0.600 (0.45)	0.618 (0.47)	1.03	0.48
2	50	0.597 (0.45)	0.728 (0.56)	1.22	0.42
2	65	0.587 (0.45)	0.676 (0.52)	1.15	0.44
2	80	0.588 (0.45)	0.643 (0.5)	1.09	0.46
2	100	0.589 (0.45)	0.612 (0.47)	1.04	0.48
4	50	0.584 (0.45)	0.661 (0.52)	1.13	0.44
4	65	0.580 (0.45)	0.618 (0.48)	1.07	0.47
4	80	0.579 (0.45)	0.592 (0.46)	1.02	0.49
4	100	0.579 (0.45)	0.565 (0.44)	0.98	0.52
6	50	0.573 (0.44)	0.625 (0.49)	1.09	0.46
6	65	0.569 (0.44)	0.586 (0.46)	1.03	0.48
6	80	0.570 (0.45)	0.563 (0.44)	0.99	0.50
6	100	0.563 (0.45)	0.538 (0.43)	0.96	0.52
8	50	0.567 (0.44)	0.614 (0.48)	1.08	0.46
8	65	0.563 (0.45)	0.569 (0.45)	1.01	0.49
8	80	0.557 (0.44)	0.544 (0.43)	0.98	0.51
8	100	0.556 (0.45)	0.524 (0.42)	0.94	0.53

Accuracy of the adjusted scores was independently influenced by both the number of linking videos and the proportion of participating examiners. Changing the number of linking videos per examiner (whilst averaging across all of the included categories of examiner participation, i.e. keeping this constant) gave error ratios for 0 video = 1.03, 2 videos = 1.13, 4 = 1.05, 6 = 1.02, 8 = 1.00, with corresponding proportions of students seeing increased score accuracy (pAcc) values of 0 videos = 0.48, 2 videos = 0.45, 4 = 0.48, 6 = 0.49, 8 = 0.50 respectively. Notably, therefore, the accuracy of adjusted scores was reduced (compared to no linking) by having 2 linking videos per examiner, but then progressively slowly increased for larger number of linking videos.

Changing the proportion of participating examiners (whilst averaging across all of the included categories of linking videos, thereby keeping those constant) showed a more linear pattern, giving error ratios for 50% of examiners = 1.11, 65% of examiners = 1.06, 80% = 1.02 and 100 of examiners = 0.99. Corresponding proportions of students whose scores became more accurate (pAcc) were 50% of examiners = 0.45, 65% examiners = 0.47, 80% = 0.49 and 100% = 0.51 respectively.

### Research question 2

How is the accuracy of VESCA score estimates influenced by:

- Differing extents of baseline differences in examiner stringency between different sites (0%, 5%, 10%, 20%)
- The number of stations in the OSCE (6, 12, or 18 stations)
- The combination of these two effects

These questions were addressed by study 2. The accuracy of adjusted scores varied substantially in this study. Error ratio (ErR) values ranged from a worst case 1.42 (i.e. adjusted scores contained 42% *more* error than observed scores) for 0% baseline difference in examiner stringency, with 18 OSCE stations, to a best case of 0.29 (i.e. score adjustment removed 71% of the error in the observed scores) for 20% difference in baseline examiner stringency with 12 OSCE stations. The proportion of students whose scores became more accurate (pAcc) as a result of adjustment showed a corresponding pattern, ranging from pAcc = 0.37 (only 37% of students’ scores became more accurate for 0% baseline difference and 18 OSCE stations, to pAcc = 0.93 (93% of students’ scores became more accurate) for 20% baseline difference and 18 OSCE stations, with a very similar finding (pAcc = 0.92) for 20% baseline difference and 12 OSCE stations. A detailed breakdown of all permutations of these parameters can be seen in Table 2.

**Table 2:** Influence of stations in the OSCE and degree of baseline difference in examiner stringency on adjusted score accuracy

Degree of baseline difference between school (% of scale)	Number of Stations in OSCE	Mean Error in Observed scores (SD)	Mean Error in Adjusted Scores (SD)	Error ratio	Proportion of students’ whose scores became more accurate through adjustment
0	6	0.814 (0.63)	0.829 (0.64)	1.02	0.49
0	12	0.579 (0.45)	0.592 (0.46)	1.02	0.49
0	18	0.475 (0.37)	0.674 (0.52)	1.42	0.37
5	6	0.907 (0.7)	0.828 (0.64)	0.91	0.54
5	12	0.712 (0.54)	0.592 (0.46)	0.83	0.59
5	18	0.635 (0.47)	0.673 (0.52)	1.06	0.49
10	6	1.172 (0.85)	0.825 (0.64)	0.70	0.67
10	12	1.056 (0.68)	0.589 (0.46)	0.56	0.75
10	18	1.026 (0.59)	0.67 (0.52)	0.65	0.70
20	6	2.012 (1.07)	0.82 (0.64)	0.41	0.85
20	12	1.996 (0.83)	0.586 (0.45)	0.29	0.92
20	18	1.998 (0.72)	0.667 (0.52)	0.33	0.93

Accuracy of the adjusted scores showed different relationships with the baseline difference in examiner stringency and the number of OSCE stations. Changing the baseline difference in examiner stringency (whilst averaging across the 3 different numbers of OSCE stations, i.e. keeping this parameter constant) gave error ratios for 0% baseline difference = 1.15, 5% baseline difference = 0.93, 10% = 0.64, and 20% = 0.34 with corresponding proportions of students seeing increased scores accuracy (pAcc) values of at 0% baseline difference = 0.45, 5% = 0.54, 10% = 0.71 and 20% = 0.90 respectively. Consequently at 0% baseline difference in examiner stringency, score adjustment made scores less accurate, whereas at 20% baseline difference in examiner stringency, 66% of error was removed and 90% of students’ scores became more accurate.

Changing the number of stations in the OSCE (whilst averaging across all levels of baseline difference in examiners stringency, thereby keeping those constant) gave error ratios for 6 OSCE stations of 0.76, 12 stations of 0.68 and 18 stations of 0.87. Corresponding proportions of students whose scores became more accurate (pAcc) were 6 stations = 0.64, 12 stations = 0.69, and 18 stations = 0.62. Consequently, these different numbers of OSCE stations produced a U-shaped influence on adjusted score accuracy, with adjustments made from an OSCE with 12 stations showing greater accuracy than the score adjustments made from either a 6 or 18 station OSCE. Notably, however, the extent of error in observed scores for 18 stations (i.e. the amount of error contained in the unadjusted scores produced by examiners) is lower than for 12 stations (3^rd^ column Table 2), so this observation may arise from an interaction of the effectiveness of score adjustment with the amount of error originally present.

### Research question 3

How is the accuracy of score estimates produced by VESCA influenced by reduction in the degree of random variability in examiners’ scoring (random error divided by 2, by 4, and by 8).

This question was addressed by study 3. As in study 1, it was performed with an assumption of 0% baseline difference between sites, and used standard parameters (12 station, 4 linking videos and 80% examiner participation). Accuracy of adjusted scores increased progressively as the amount of random error was reduced. Error ratios (ErR) for the usual extent of random examiner error = 1.02, half usual random examiner error (err/2) = 0.86, one quarter random error (err/4) = 0.69, and one eighth usual random examiner error (err/8) was 0.59. corresponding proportions of students whose scores became more accurate were: usual examiner error = 0.49, err/2 = 0.56, err/4 = 0.62, err /8 = 0.66. Consequently, whilst reducing the degree of modelled random error within examiners’ scoring increased accuracy, a very substantial reduction in examiners’ random error (one eighth its usual value) produced a moderate increase in accuracy (41% reduction in error; 66% of students’ scores became more accurate). A detailed breakdown of these data are available in Table 3.

**Table 3:** Influence of reduction in examiner random error on adjusted score accuracy

Reduction in error	Mean Error in Observed scores (SD)	Mean Error in Adjusted Scores (SD)	Error ratio	Proportion of students’ whose scores became more accurate through adjustment
Error / 2	0.375 (0.29)	0.323 (0.25)	0.86	0.56
Error / 4	0.302 (0.24)	0.207 (0.16)	0.69	0.62
Error / 8	0.28 (0.22)	0.166 (0.13)	0.59	0.66

### Research question 4

How does the proportion of candidates whose scores become more accurate vary for different sizes of score adjustment for each of the parameters investigated within RQs 1–2.

This study produced 32 tables of tabulated results. These findings, along with summary text and further details of how they were calculated, are presented in appendix 1. In summary, when there was no baseline difference between sites (i.e. study 1) the findings did not demonstrate a threshold for any of the studied parameters beyond which the target of pAcc > 0.8 was achieved. Notably the vast majority of adjustments made in study 1 were comparatively small. When larger baseline differences existed (10–20% baseline difference, see study 2) adjustments were typically larger, with a majority exceeding 9% of the assessment scale for 20% baseline differences. Thresholds in the region of 3–4% of the assessment scale could be set for scenarios where a baseline difference of 20% existed, to achieve a target of pAcc > 0.8. Notably, therefore adjustment thresholds depended on the degree of baseline difference rather than an absolute value of the adjustment threshold.

## Discussion

### Summary of results

This study has produced several novel insights into the accuracy of score adjustments produced by VESCA under a range of plausible OSCE conditions. As VESCA is an example of adjusting raters’ scores by MFRM based on limited linking, these findings may also have broader applicability to rater-based judgements more generally. Firstly, our study has shown that VESCA can substantially increase score accuracy when there are large differences between the average standard of examiners in different sites. Under typical operating conditions (i.e. 12 stations, 4 linking videos per examiners, 80% examiner participation), when there was 20% baseline difference between the stringency of examiners at different sites, score adjustment became very much more accurate than unadjusted scores, reducing error by 59–73% and resulting in 85–93% of students’ scores becoming more accurate. Consequently, VESCA was able to have a substantial benefit in this scenario.

Conversely, in the absence of systematic differences between parallel groups of examiners, score adjustment overall made scores *less* accurate than the observed scores produced by examiners, with an increase in the total error and only a minority of students’ scores becoming more accurate. This latter finding is surprising and unexpected. Next, this study has shown that the accuracy of adjusted scores is indeed sensitive to the theorised parameters of the number of linking video per examiner, and the proportion of examiners who participate, but within what we expect to be reasonable limits, increasing these parameters only modestly increased the accuracy of the resulting score adjustments. Lastly, the study has shown that the accuracy of adjusted scores can be increased by reducing examiners’ random error variability, but that a substantial reduction in this error (i.e. 1/8^th^ its usual extent) is required to produce a moderate (41%) reduction in error, making 66% of students’ scores more accurate.

### Theoretical considerations

Whilst Many Facet Rasch Modelling can be used for a number of purposes, one of its explicit intended applications is to place disparate groups of examiners on a common scale (or within a single frame of reference) by linking and equating for their differences [31]. Consequently, whilst VESCA’s processes within OSCEs are comparatively novel, the process of using limited linkage to equate for examiner differences using Many Facet Rash Modelling is not. Indeed, established guidance on using Many Facet Rasch Modelling provides consideration of different linking patterns, including more sparse linkage patterns than we employed in this study [34]. None of this material suggests that there are circumstances in which adjusted scores produced by FACETS will become less accurate than the original scores, so some readers may be surprised to see that this occurred to some extent in all of our scenarios and was very frequent in all situations where there were no baseline differences between sites. This may be because the extent of random variance in OSCEs is comparatively large [22]. From a theoretical perspective, it appears that Many Facet Rasch Modelling becomes increasingly robust when there are progressively larger systematic differences to account for, and when there is less random error. Conversely, stronger linking through greater proportions of examiner participation or more linking videos produces on modest improvements in accuracy. Essentially, Many Facet Rasch Modelling (and by extension VESCA) is useful for adjusting when systematic differences are substantially larger than random variability but performs poorly when systematic differences are small relative to random error. Critically, therefore, practitioners who seek to use it to adjust scores need to know when there are large systematic differences between groups of examiners.

### Practical recommendation

Establishing the extent of systematic difference between sites or examiner-cohorts is in most conventional distributed OSCEs is extremely difficult, as observed scores confound the combined influence of student ability and examiner stringency, meaning that observed scores may mask differences in examiner stringency, or conversely that observed differences may arise due to genuine differences in students’ performance. By asking examiners to score station specific videos, VESCA provides controlled comparisons of examiners’ scoring on a subset of the examined scores and are therefore directly applicable to the task in hand. Given that baseline differences may therefore be undetected by conventional approaches, we recommend that there may be benefit for organisations who run large distributed OSCEs to use the first 2 steps of VESCA in practice (1/ filming videos and 2/ asking examiners to score them) to monitor for baseline differences between sites. This would allow the scores allocated to videos by different groups of examiners to be directly compared as part of quality assurance procedures. The regularity of such monitoring could be varied depending on the degree of baseline examiner differences which are observed and the stakes of the OSCE and resources of the organisation. Where feasible, these comparisons should be made before results are released, to enable the option to adjust scores based on these findings. Previous uses of VESCA have allowed 2–3 weeks for examiners to score videos [19, 35], with roughly a further week required for data alignment and analysis. Therefore, we accept that this recommendation has the potential to delay release of results for some organisations.

Having used step 1 & 2 of VESCA to monitor inter-site examiner differences, where examiner score comparisons suggest negligible differences, faculty should be reassured and the analysis is expected to produce useful quality assurance information. Where findings suggest 5–10% inter-site differences, the evidence we have provided here suggests that rather than using adjusted scores, faculty should then focus faculty development efforts on sites or groups of examiners where scoring was discordant, but they should continue to use the original score. If video scores suggest a baseline difference between sites in the region of 10–20% of the assessment scale, then faculty may consider using the adjusted scores instead of raw scores as these will substantially reduce error and will increase score accuracy for the overwhelming majority of students.

Performing the VESCA procedures requires a non-trivial investment of time and effort by faculty and our results suggest it is only likely to be worthwhile when large systematic differences occur. Concordantly, it is pertinent to consider how likely this situation is in practice. The simplest answer to this question may be that as they are rarely measured, we do not know. Sebok et al. [5], however, attributed up to 17% of observed score variance to examiners in different sites in a national distributed examine, although this occurred for only a minority of occasions examined. Whilst comparing standard setting for knowledge testing, Taylor 2017 et al. [36] found up to 25% points difference between different schools who set the highest and lowest standards for the same items. More recently, Yeates et al., using the VESCA methodology, showed a 16.3% difference between the average standard of examiners’ judgements in a graduation-level formative OSCE which was shared between four UK medical schools [37]. Consequently, it is clear that large site-based variations can occur in assessment in some instances, and therefore it seems both prudent to monitor for their occurrence and reassuring to know that adjustment can be dependable when differences are substantial.

### Limitations

We believe that our study had numerous strengths. We based our simulation on parameters from real data, modelling plausible scenarios and using a rigours step-wise approach to data modelling which we assert is expected to have produced a plausible imitation of reality. Despite this, our study has some limitations. All simulation is limited by the parameters of the simulation. In this study, we modelled all known substantial influences on OSCE scores (candidate, station, examiner, and appropriate random variance terms) [21, 22, 38], but omitted influences shown more recently to be minor such as contrast effects or differential rater function over time [39]. Importantly, we can’t comment on combinations of parameters which we didn’t test (for example 60% examiner participation, 3 linking videos or 12% baseline difference) nor can we infer beyond the range of modelled parameters (i.e. 12 linking videos). None the less, we assert that the modelled parameters represent a realistic range of likely use.

Each simulation only modelled 60 students. This value was chosen for computational simplicity. Adding additional students would have required a greater number of examiner groups, but would not have increased their distribution beyond the specified range in each simulation, so is unlikely to have produced different results.

Our modelling made unidimensional assumptions. Multi-dimensionality in the data could theoretically have further attenuated accuracy. OSCE data examined within prior uses of VESCA have been unidimensional [17–19] and as data dimensionality should be checked before MFRM is used, we assert that this assumption was reasonable.

### Future research

As with all modelling, these data would benefit from independent replication by a different group adopting a different approach. Further research could determine VESCA’s accuracy in some of the scenarios we didn’t test, for example a 15% baseline difference with either 100% or only 65% examiner participation, in order to extend our understanding of when it is reasonable to use adjusted scores in practice. Additional research should seek to determine whether any general relationship exists between 1/ degree of linking, 2/ size of baseline difference and 3/ degree of random error on the accuracy of score adjustment made by Many facet Rasch modelling. Lastly, it would be helpful to explore whether the potential for unmeasured inter-site examiner differences can be predicted without the need for VESCA using rater x site interactions within Generalizability analyses. If such analysis can predict inter-site differences, this would guide organisations to decide when it may be helpful to employ the VESCA procedures.

## Conclusion

The accuracy of score adjustment produced by VESCA under typical operating conditions, when there are no baseline differences between examiner groups, is low and we do not support the use of adjusted scores from VESCA under these circumstances. Conversely, when large baseline differences exist between locations, score adjustment becomes substantially more accurate and consideration could be given to using VESCA-adjusted scores in these scenarios. By comparing examiners’ scoring of videos, VESCA provides directly relevant controlled comparisons of the influence of different examiner groups from different locations within distributed OSCE. These findings offer a basis to support its use in practice within defined parameters.

## Supplementary Information

Supplementary Material 1

## Data Availability

computer code used for generation and analysis available on request by contacting Peter Yeates at p.yeates@keele.ac.uk
