# Supplementary material for: Investigating the accuracy of adjusting for examiner differences in multi-centre Objective Structured Clinical Exams (OSCEs). A simulation study of video-based Examiner Score Comparison and Adjustment (VESCA)
Source: BMC Med Educ. 2024 Dec 18;24:1466. doi: 10.1186/s12909-024-06462-3 (PMC11654327; doi:10.1186/s12909-024-06462-3)
Supplement: Supplementary file 1 — Supplementary Material 1 [file 12909_2024_6462_MOESM1_ESM.pdf]

Appendix 1

Descriptive summary of adjustment threshold results

Study 1:

For all parameters examined in study 1, the vast majority of score adjustments were small, with between 74-94% of score adjustments <4%.

In all scenarios with 0 or 2 linking videos, and in the 4 linking video scenarios with 50%, 65% and 80% examiner participation, imposing an adjustment threshold would either not improve or worsen the resulting proportionate accuracy of score adjustments, with values never exceeding a pAcc of 0.5, therefore well short of the desired pAcc of 0.8

For 4 linking videos with 100% examiner participation, and for scenarios with 6 or 8 linking videos, some categories of score adjustment size showed slightly higher pAcc values, in the range of 0.50-0.63. There was, however, no clear pattern of improvement in the proportionate accuracy of adjustments across successive categories of adjustment size: in some individual scenarios a threshold would have improved adjustment accuracy (for example 8 linking videos with 80% examiner participation, where an adjustment threshold of 3% would increase accuracy from an average value of 0.51 to between 0.52-0.57), whereas in others this was not the case, as the pattern either fluctuated across categories, or proportionate accuracy declined for later categories. Regardless, where thresholds appeared to improve accuracy, these improvements only applied to very small numbers of students. No scenarios occurred in which a threshold could be set, above which a pAcc of >0.8 would be achieved.

Study 2:

Consistent with the findings of study 1, when there was 0% baseline difference between schools, there was no adjustment threshold above which proportionate accuracy was improved, regardless of the number of stations in the OSCE. pAcc values showed a maximum of 0.52. The majority of adjustments were small, with 74-88% of adjustments <4% of the assessment scale.

The size of score adjustments progressively increased for larger baseline differences. For scenarios with 5% baseline difference between schools, between 63-72% of students score adjustments were <4% of the assessment scale; for 10% baseline difference, between 36-42% of students' score adjustment were <4%, with corresponding increases in the frequency of larger score adjustments.

For both 5% and 10% baseline differences between schools, pAcc values tended to increase for progressively larger adjustment threshold values, reaching pAcc values in the region of 0.63-0.67 for 5% baseline difference and in the region of 0.71-0.78 for 10% baseline difference. Conversely when the modelled OSCEs had 18 stations, at both 5% and 10% baseline difference between schools, the opposite was observed, with larger adjustments showing lower accuracies than smaller adjustments, and overall pAcc values peaking at 0.51 for 18 stations, 5% baseline difference, and 0.78 for 18 stations, 10% baseline difference. As a result, whilst some of these pAcc values approached the target of 0.8, the inconsistent findings across different numbers of OSCE stations makes it difficult to confidently support the use of an adjustment threshold.

Score adjustments were generally large in scenarios where there was a 20% baseline difference between schools, with 59-66% of students receiving score adjustments >9% of the assessment scale.

When there was a 20% baseline difference between schools, pAcc values of >0.8 were achieved for all numbers of OSCE stations. For both 6 and 12 station OSCEs, setting an adjustment threshold of 4% would have ensured that all score adjustments had pAcc values > 0.8; for an 18 station OSCE, setting an adjustment threshold of 3% would have ensured that all score adjustments had pAcc values > 0.8. Notably in these scenarios, very small numbers of students were excluded from score adjustments by using these thresholds as the majority of students received larger adjustments.

These data indicate that no absolute adjustment threshold could be defined from these data above which a pAcc of >0.8 would consistently be achieved. Instead, the ability to set thresholds was dependent on the degree of baseline difference.

Study 1: Proportions of students receiving each category of score adjustment / change in accuracy

No linking Videos, 50% Examiner participation

		Size of score adjustment										
Change in score accuracy		0-1%	1-2%	2-3%	3-4%	4-5%	5-6%	6-7%	7-8%	8-9%	>9%	Overall
Worse	>6%	0	0	0	0	0	0	0	0	0	0	0.00
	4-6%	0	0	0	0	0	0.003	0.002	0.001	0	0	0.01
	2-4%	0	0	0.028	0.035	0.013	0.002	0.001	0	0	0	0.08
	0-2%	0.204	0.146	0.066	0.011	0.004	0.002	0.001	0	0	0	0.43
	0-2%	0.195	0.138	0.058	0.011	0.004	0.002	0.001	0	0	0	0.41
	2-4%	0	0	0.021	0.028	0.012	0.001	0.001	0	0	0	0.06
	4-6%	0	0	0	0	0	0.003	0.001	0	0	0	0.00
Better	>6%	0	0	0	0	0	0	0	0	0	0	0.00
Proportion better		0.49	0.49	0.46	0.46	0.48	0.46	0.43	0.00	0.00	0.00	0.48
Frequency		0.399	0.284	0.173	0.085	0.033	0.013	0.007	0.001	0.000	0.000	
Cumulitive Frequency		0.399	0.683	0.856	0.941	0.974	0.987	0.994	0.995	0.995	0.995	

No linking Videos, 65% Examiner participation

		Size of score adjustment										
Change in score accuracy		0-1%	1-2%	2-3%	3-4%	4-5%	5-6%	6-7%	7-8%	8-9%	>9%	Overall
Worse	>6%	0	0	0	0	0	0	0	0	0	0	0.00
	4-6%	0	0	0	0	0	0.003	0.001	0.001	0	0	0.01
	2-4%	0	0	0.029	0.034	0.016	0.002	0.001	0	0	0	0.08
	0-2%	0.197	0.152	0.058	0.012	0.005	0.002	0.001	0	0	0	0.43
	0-2%	0.191	0.141	0.056	0.011	0.005	0.002	0.001	0	0	0	0.41
	2-4%	0	0	0.024	0.029	0.015	0.002	0.001	0	0	0	0.07
	4-6%	0	0	0	0	0	0.003	0.001	0	0	0	0.00
Better	>6%	0	0	0	0	0	0	0	0	0	0	0.00
Proportion more accurate		0.49	0.48	0.48	0.47	0.49	0.50	0.50	0.00	0.00	0.00	0.48
Frequency		0.388	0.293	0.167	0.086	0.041	0.014	0.006	0.001	0.000	0.000	
Cumulitive Frequency		0.388	0.681	0.848	0.934	0.975	0.989	0.995	0.996	0.996	0.996	

No linking Videos, 80% Examiner participation

		Size of score adjustment										
Change in score accuracy		0-1%	1-2%	2-3%	3-4%	4-5%	5-6%	6-7%	7-8%	8-9%	>9%	Overall
Worse	>6%	0	0	0	0	0	0	0	0	0	0	0.00
	4-6%	0	0	0	0	0	0.004	0.002	0	0	0	0.01
	2-4%	0	0	0.029	0.034	0.016	0.002	0.001	0	0	0	0.08
	0-2%	0.189	0.152	0.061	0.012	0.005	0.002	0.001	0	0	0	0.42
	0-2%	0.189	0.144	0.056	0.012	0.006	0.002	0.001	0	0	0	0.41
	2-4%	0	0	0.026	0.031	0.015	0.002	0.001	0	0	0	0.08
	4-6%	0	0	0	0	0	0.003	0.001	0	0	0	0.00
Better	>6%	0	0	0	0	0	0	0	0	0	0	0.00
Proportion more accurate		0.50	0.49	0.48	0.48	0.50	0.47	0.43	0.00	0.00	0.00	0.49
Frequency		0.378	0.296	0.172	0.089	0.042	0.015	0.007	0.000	0.000	0.000	
Cumulitive Frequency		0.378	0.674	0.846	0.935	0.977	0.992	0.999	0.999	0.999	0.999	

No linking Videos, 100% Examiner participation

Size of score adjustment

**Change in score accuracy**		0-1%	1-2%	2-3%	3-4%	4-5%	5-6%	6-7%	7-8%	8-9%	>9%	Overall
**Worse**	>6%	0	0	0	0	0	0	0	0	0	0	0.00
	4-6%	0	0	0	0	0	0.004	0.002	0.001	0	0	0.01
	2-4%	0	0	0.028	0.033	0.015	0.002	0	0	0	0	0.08
	0-2%	0.201	0.156	0.056	0.011	0.004	0.002	0.001	0	0	0	0.43
	0-2%	0.195	0.143	0.054	0.01	0.005	0.002	0.001	0	0	0	0.41
	2-4%	0	0	0.024	0.025	0.013	0.002	0.001	0	0	0	0.07
	4-6%	0	0	0	0	0	0.003	0.001	0	0	0	0.00
**Better**	>6%	0	0	0	0	0	0	0	0	0	0	0.00
Proportion more accurate		0.49	0.48	0.48	0.44	0.49	0.47	0.50	0.00	0.00	0.00	**0.48**
Frequency		0.396	0.299	0.162	0.079	0.037	0.015	0.006	0.001	0.000	0.000	
Cumulitive Frequency		0.396	0.695	0.857	0.936	0.973	0.988	0.994	0.995	0.995	0.995	

Two linking video, 50% Examiner Participation

Size of score adjustment

**Change in score accuracy**		0-1%	1-2%	2-3%	3-4%	4-5%	5-6%	6-7%	7-8%	8-9%	>9%	
**Worse**	>6%	0	0	0	0	0	0	0	0.003	0.005	0.006	0.01
	4-6%	0	0	0	0	0.001	0.026	0.016	0.006	0.002	0.002	0.05
	2-4%	0	0	0.033	0.062	0.052	0.008	0.005	0.003	0.002	0.002	0.17
	0-2%	0.121	0.116	0.061	0.019	0.014	0.009	0.005	0.003	0.002	0.001	0.35
	0-2%	0.11	0.098	0.047	0.017	0.013	0.007	0.005	0.002	0.001	0.001	0.30
	2-4%	0	0	0.023	0.037	0.027	0.006	0.003	0.002	0.001	0.001	0.10
	4-6%	0	0	0	0	0	0.009	0.004	0.002	0.001	0	0.02
**Better**	>6%	0	0	0	0	0	0	0	0	0.001	0	0.00
Prop better		0.48	0.46	0.43	0.40	0.37	0.34	0.32	0.29	0.27	0.15	**0.42**
Freq		0.231	0.214	0.164	0.135	0.107	0.065	0.038	0.021	0.015	0.013	
Cumulit Freq		0.231	0.445	0.609	0.744	0.851	0.916	0.954	0.975	0.99	1.003	

Two linking video, 65% Examiner Participation

Size of score adjustment

**Change in score accuracy**		0-1%	1-2%	2-3%	3-4%	4-5%	5-6%	6-7%	7-8%	8-9%	>9%	
**Worse**	>6%	0	0	0	0	0	0	0	0.002	0.002	0.002	0.01
	4-6%	0	0	0	0	0	0.018	0.013	0.003	0.001	0.001	0.04
	2-4%	0	0	0.035	0.062	0.04	0.006	0.004	0.002	0.001	0.001	0.15
	0-2%	0.14	0.122	0.062	0.018	0.012	0.007	0.004	0.002	0.001	0	0.37
	0-2%	0.131	0.105	0.052	0.018	0.011	0.006	0.003	0.002	0.001	0	0.33
	2-4%	0	0	0.024	0.039	0.023	0.004	0.003	0.001	0.001	0	0.10
	4-6%	0	0	0	0	0	0.007	0.004	0.002	0	0	0.01
**Better**	>6%	0	0	0	0	0	0	0	0	0.001	0	0.00
Prop better		0.48	0.46	0.44	0.42	0.40	0.35	0.32	0.36	0.38	0.00	**0.44**
Freq		0.271	0.227	0.173	0.137	0.086	0.048	0.031	0.014	0.008	0.004	
Cumulit Freq		0.271	0.498	0.671	0.808	0.894	0.942	0.973	0.987	0.995	0.999	

Two linking video, 80% Examiner Participation

Change in score accuracy		Size of score adjustment 0-1%	1-2%	2-3%	3-4%	4-5%	5-6%	6-7%	7-8%	8-9%	>9%	
Worse	>6%	0	0	0	0	0	0	0	0.001	0.001	0.001	0.00
	4-6%	0	0	0	0	0	0.015	0.007	0.003	0.001	0.001	0.03
	2-4%	0	0	0.036	0.052	0.034	0.005	0.003	0.001	0	0	0.13
	0-2%	0.153	0.125	0.06	0.018	0.01	0.006	0.003	0.001	0	0.001	0.38
	0-2%	0.145	0.114	0.052	0.017	0.01	0.005	0.003	0.001	0	0	0.35
	2-4%	0	0	0.025	0.04	0.022	0.004	0.002	0.001	0	0	0.09
	4-6%	0	0	0	0	0	0.008	0.004	0.001	0	0	0.01
Better	>6%	0	0	0	0	0	0	0	0	0	0	0.00
Prop better		0.49	0.48	0.45	0.45	0.42	0.40	0.41	0.33	0.00	0.00	**0.46**
Freq		0.298	0.239	0.173	0.127	0.076	0.043	0.022	0.009	0.002	0.003	
Cumulit Freq		0.298	0.537	0.71	0.837	0.913	0.956	0.978	0.987	0.989	0.992	

Two linking video, 100% Examiner Participation

Change in score accuracy		Size of score adjustment 0-1%	1-2%	2-3%	3-4%	4-5%	5-6%	6-7%	7-8%	8-9%	>9%	
Worse	>6%	0	0	0	0	0	0	0	0	0	0	0.00
	4-6%	0	0	0	0	0	0.01	0.003	0.001	0	0	0.01
	2-4%	0	0	0.034	0.045	0.026	0.004	0.001	0.001	0	0	0.11
	0-2%	0.164	0.133	0.062	0.016	0.009	0.004	0.001	0.001	0	0	0.39
	0-2%	0.162	0.126	0.058	0.016	0.009	0.004	0.001	0	0	0	0.38
	2-4%	0	0	0.028	0.039	0.021	0.004	0.001	0	0	0	0.09
	4-6%	0	0	0	0	0	0.007	0.002	0.001	0	0	0.01
Better	>6%	0	0	0	0	0	0	0	0	0	0	0.00
Prop better		0.50	0.49	0.47	0.47	0.46	0.45	0.44	0.25	0.00	0.00	**0.48**
Freq		0.33	0.26	0.18	0.12	0.07	0.03	0.01	0.00	0.00	0.00	
Cumulit Freq		0.326	0.585	0.767	0.883	0.948	0.981	0.99	0.994	0.994	0.994	

Four linking videos, 50% Examiner Participation

Change in score accuracy		Size of score adjustment 0-1%	1-2%	2-3%	3-4%	4-5%	5-6%	6-7%	7-8%	8-9%	>9%	
Worse	>6%	0	0	0	0	0	0	0	0.002	0.002	0.002	0.01
	4-6%	0	0	0	0	0	0.015	0.009	0.004	0.001	0.001	0.03
	2-4%	0	0	0.036	0.056	0.036	0.006	0.003	0.001	0.001	0	0.14
	0-2%	0.147	0.126	0.065	0.018	0.011	0.006	0.003	0.001	0.001	0.001	0.38
	0-2%	0.136	0.109	0.057	0.017	0.009	0.006	0.002	0.001	0.001	0	0.34
	2-4%	0	0	0.025	0.038	0.022	0.004	0.002	0.001	0.001	0	0.09
	4-6%	0	0	0	0	0	0.007	0.003	0.001	0	0	0.01
Better	>6%	0	0	0	0	0	0	0	0	0	0	0.00
Prop better		0.48	0.46	0.45	0.43	0.40	0.39	0.32	0.27	0.29	0.00	**0.44**
Freq		0.283	0.235	0.183	0.129	0.078	0.044	0.022	0.011	0.007	0.004	
Cumulit Freq		0.283	0.518	0.701	0.83	0.908	0.952	0.974	0.985	0.992	0.996	

Four linking videos, 65% Examiner Participation

Change in score accuracy		0-1%	1-2%	2-3%	3-4%	4-5%	5-6%	6-7%	7-8%	8-9%	>9%	
		Size of score adjustment										
Worse	>6%	0	0	0	0	0	0	0	0.001	0.001	0.001	0.00
	4-6%	0	0	0	0	0	0.011	0.005	0.002	0	0	0.02
	2-4%	0	0	0.032	0.051	0.03	0.005	0.002	0.001	0	0	0.12
	0-2%	0.163	0.129	0.059	0.017	0.01	0.005	0.002	0.001	0	0	0.39
	0-2%	0.156	0.115	0.056	0.017	0.009	0.005	0.002	0.001	0	0	0.36
	2-4%	0	0	0.026	0.04	0.022	0.004	0.001	0.001	0	0	0.09
	4-6%	0	0	0	0	0	0.007	0.003	0.001	0	0	0.01
Better	>6%	0	0	0	0	0	0	0	0	0	0	0.00
Prop better		0.49	0.47	0.47	0.46	0.44	0.43	0.40	0.38	0.00	0.00	**0.47**
Freq		0.319	0.244	0.173	0.125	0.071	0.037	0.015	0.008	0.001	0.001	
Cumulit Freq		0.319	0.563	0.736	0.861	0.932	0.969	0.984	0.992	0.993	0.994	

Four linking videos, 80% Examiner Participation

Change in score accuracy		0-1%	1-2%	2-3%	3-4%	4-5%	5-6%	6-7%	7-8%	8-9%	>9%	
		Size of score adjustment										
Worse	>6%	0	0	0	0	0	0	0	0	0.001	0	0.00
	4-6%	0	0	0	0	0	0.008	0.003	0.001	0	0	0.01
	2-4%	0	0	0.031	0.039	0.025	0.004	0.001	0.001	0	0	0.10
	0-2%	0.18	0.132	0.058	0.014	0.008	0.004	0.001	0	0	0	0.40
	0-2%	0.174	0.125	0.055	0.015	0.009	0.004	0.001	0	0	0	0.38
	2-4%	0	0	0.027	0.037	0.022	0.004	0.001	0	0	0	0.09
	4-6%	0	0	0	0	0	0.006	0.002	0.001	0	0	0.01
Better	>6%	0	0	0	0	0	0	0	0	0	0	0.00
Prop better		0.49	0.49	0.48	0.50	0.48	0.47	0.44	0.33	0.00	0.00	**0.49**
Freq		0.354	0.257	0.171	0.105	0.064	0.030	0.009	0.003	0.001	0.000	
Cumulit Freq		0.354	0.611	0.782	0.887	0.951	0.981	0.99	0.993	0.994	0.994	

Four linking videos, 100% Examiner Participation

Change in score accuracy		0-1%	1-2%	2-3%	3-4%	4-5%	5-6%	6-7%	7-8%	8-9%	>9%	
		Size of score adjustment										
Worse	>6%	0	0	0	0	0	0	0	0	0	0	0.00
	4-6%	0	0	0	0	0	0.003	0.001	0	0	0	0.00
	2-4%	0	0	0.026	0.032	0.014	0.002	0	0	0	0	0.07
	0-2%	0.191	0.134	0.057	0.014	0.006	0.002	0.001	0	0	0	0.41
	0-2%	0.194	0.143	0.062	0.013	0.005	0.002	0.001	0	0	0	0.42
	2-4%	0	0	0.028	0.038	0.018	0.002	0.001	0	0	0	0.09
	4-6%	0	0	0	0	0	0.004	0.002	0	0	0	0.01
Better	>6%	0	0	0	0	0	0	0	0	0	0	0.00
Prop better		0.50	0.52	0.52	0.53	0.53	0.53	0.67	0.00	0.00	0.00	**0.52**
Freq		0.385	0.277	0.173	0.097	0.043	0.015	0.006	0.000	0.000	0.000	
Cumulit Freq		0.385	0.662	0.835	0.932	0.975	0.99	0.996	0.996	0.996	0.996	

Six linking videos, 50% Examiner Participation

Size of score adjustment

**Change in score accuracy**		0-1%	1-2%	2-3%	3-4%	4-5%	5-6%	6-7%	7-8%	8-9%	>9%	
**Worse**	>6%	0	0	0	0	0	0	0	0.001	0.001	0.001	0.00
	4-6%	0	0	0	0	0	0.013	0.006	0.003	0	0	0.02
	2-4%	0	0	0.032	0.049	0.033	0.005	0.003	0.001	0	0.001	0.12
	0-2%	0.162	0.133	0.06	0.017	0.01	0.005	0.003	0.001	0	0	0.39
	0-2%	0.152	0.117	0.051	0.016	0.01	0.005	0.003	0.001	0	0	0.36
	2-4%	0	0	0.024	0.037	0.023	0.004	0.002	0.001	0	0	0.09
	4-6%	0	0	0	0	0	0.007	0.003	0.001	0	0	0.01
**Better**	>6%	0	0	0	0	0	0	0	0	0	0	0.00
Prop better		0.48	0.47	0.45	0.45	0.43	0.41	0.40	0.33	0.00	0.00	**0.46**
Freq		0.314	0.250	0.167	0.119	0.076	0.039	0.020	0.009	0.001	0.002	
Cumulit Freq		0.314	0.564	0.731	0.85	0.926	0.965	0.985	0.994	0.995	0.997	

Six linking videos, 65% Examiner Participation

Size of score adjustment

**Change in score accuracy**		0-1%	1-2%	2-3%	3-4%	4-5%	5-6%	6-7%	7-8%	8-9%	>9%	
**Worse**	>6%	0	0	0	0	0	0	0	0	0	0	0.00
	4-6%	0	0	0	0	0	0.007	0.003	0.001	0	0	0.01
	2-4%	0	0	0.031	0.044	0.022	0.003	0.001	0.001	0	0	0.10
	0-2%	0.177	0.136	0.062	0.015	0.007	0.003	0.002	0.001	0	0	0.40
	0-2%	0.174	0.125	0.06	0.014	0.008	0.003	0.001	0.001	0	0	0.39
	2-4%	0	0	0.027	0.037	0.019	0.003	0.001	0.001	0	0	0.09
	4-6%	0	0	0	0	0	0.005	0.002	0.001	0	0	0.01
**Better**	>6%	0	0	0	0	0	0	0	0	0	0	0.00
Prop better		0.50	0.48	0.48	0.46	0.48	0.46	0.40	0.50	0.00	0.00	**0.48**
Freq		0.351	0.261	0.180	0.110	0.056	0.024	0.010	0.006	0.000	0.000	
Cumulit Freq		0.351	0.612	0.792	0.902	0.958	0.982	0.992	0.998	0.998	0.998	

Six linking videos, 80% Examiner Participation

Size of score adjustment

**Change in score accuracy**		0-1%	1-2%	2-3%	3-4%	4-5%	5-6%	6-7%	7-8%	8-9%	>9%	
**Worse**	>6%	0	0	0	0	0	0	0	0	0	0	0.00
	4-6%	0	0	0	0	0	0.004	0.001	0	0	0	0.01
	2-4%	0	0	0.025	0.033	0.018	0.002	0	0	0	0	0.08
	0-2%	0.196	0.137	0.054	0.014	0.007	0.002	0.001	0	0	0	0.41
	0-2%	0.193	0.143	0.055	0.014	0.007	0.002	0.001	0	0	0	0.42
	2-4%	0	0	0.025	0.035	0.02	0.002	0.001	0	0	0	0.08
	4-6%	0	0	0	0	0	0.004	0.001	0	0	0	0.01
**Better**	>6%	0	0	0	0	0	0	0	0	0	0	0.00
Prop better		0.50	0.51	0.50	0.51	0.52	0.50	0.60	0.00	0.00	0.00	**0.50**
Freq		0.389	0.280	0.159	0.096	0.052	0.016	0.005	0.000	0.000	0.000	
Cumulit Freq		0.389	0.669	0.828	0.924	0.976	0.992	0.997	0.997	0.997	0.997	

Six linking videos, 100% Examiner Participation

Size of score adjustment

**Change in score accuracy**		0-1%	1-2%	2-3%	3-4%	4-5%	5-6%	6-7%	7-8%	8-9%	>9%	
**Worse**	>6%	0	0	0	0	0	0	0	0	0	0	0.00
	4-6%	0	0	0	0	0	0.002	0.001	0	0	0	0.00
	2-4%	0	0	0.023	0.027	0.009	0.001	0	0	0	0	0.06
	0-2%	0.211	0.126	0.055	0.011	0.005	0.002	0	0	0	0	0.41
	0-2%	0.217	0.137	0.065	0.012	0.005	0.001	0	0	0	0	0.44
	2-4%	0	0	0.027	0.036	0.015	0.002	0	0	0	0	0.08
	4-6%	0	0	0	0	0	0.003	0.001	0	0	0	0.00
**Better**	>6%	0	0	0	0	0	0	0	0	0	0	0.00
Prop better		0.51	0.52	0.54	0.56	0.59	0.55	0.50	0.00	0.00	0.00	**0.52**
Freq		0.428	0.263	0.170	0.086	0.034	0.011	0.002	0.000	0.000	0.000	
Cumulit Freq		0.428	0.691	0.861	0.947	0.981	0.992	0.994	0.994	0.994	0.994	

Eight linking videos, 50% Examiner Participation

Size of score adjustment

**Change in score accuracy**		0-1%	1-2%	2-3%	3-4%	4-5%	5-6%	6-7%	7-8%	8-9%	>9%	
**Worse**	>6%	0	0	0	0	0	0	0	0.001	0.001	0.001	0.00
	4-6%	0	0	0	0	0	0.012	0.006	0.002	0	0	0.02
	2-4%	0	0	0.031	0.045	0.028	0.005	0.002	0.001	0	0	0.11
	0-2%	0.168	0.139	0.065	0.015	0.01	0.005	0.002	0.001	0.001	0	0.41
	0-2%	0.157	0.12	0.053	0.014	0.009	0.005	0.002	0.001	0	0	0.36
	2-4%	0	0	0.024	0.035	0.021	0.004	0.001	0.001	0	0	0.09
	4-6%	0	0	0	0	0	0.007	0.002	0	0	0	0.01
**Better**	>6%	0	0	0	0	0	0	0	0	0	0	0.00
Prop better		0.48	0.46	0.45	0.45	0.44	0.42	0.33	0.29	0.00	0.00	**0.46**
Freq		0.325	0.259	0.173	0.109	0.068	0.038	0.015	0.007	0.002	0.001	
Cumulit Freq		0.325	0.584	0.757	0.866	0.934	0.972	0.987	0.994	0.996	0.997	

Eight linking videos, 65% Examiner Participation

Size of score adjustment

**Change in score accuracy**		0-1%	1-2%	2-3%	3-4%	4-5%	5-6%	6-7%	7-8%	8-9%	>9%	
**Worse**	>6%	0	0	0	0	0	0	0	0	0	0	0.00
	4-6%	0	0	0	0	0	0.005	0.002	0.001	0	0	0.01
	2-4%	0	0	0.03	0.039	0.018	0.002	0.001	0	0	0	0.09
	0-2%	0.191	0.133	0.058	0.015	0.007	0.003	0.001	0	0	0	0.41
	0-2%	0.188	0.126	0.057	0.015	0.007	0.003	0.001	0	0	0	0.40
	2-4%	0	0	0.028	0.037	0.019	0.003	0.001	0	0	0	0.09
	4-6%	0	0	0	0	0	0.004	0.001	0	0	0	0.01
**Better**	>6%	0	0	0	0	0	0	0	0	0	0	0.00
Prop better		0.50	0.49	0.49	0.49	0.51	0.50	0.43	0.00	0.00	0.00	**0.49**
Freq		0.379	0.259	0.173	0.106	0.051	0.020	0.007	0.001	0.000	0.000	
Cumulit Freq		0.379	0.638	0.811	0.917	0.968	0.988	0.995	0.996	0.996	0.996	

Eight linking videos, 80% Examiner Participation

Size of score adjustment

**Change in score accuracy**		0-1%	1-2%	2-3%	3-4%	4-5%	5-6%	6-7%	7-8%	8-9%	>9%	
**Worse**	>6%	0	0	0	0	0	0	0	0	0	0	0.00
	4-6%	0	0	0	0	0	0.003	0.001	0	0	0	0.00
	2-4%	0	0	0.024	0.03	0.012	0.002	0.001	0	0	0	0.07
	0-2%	0.212	0.132	0.055	0.012	0.005	0.002	0.001	0	0	0	0.42
	0-2%	0.206	0.134	0.058	0.013	0.005	0.002	0.001	0	0	0	0.42
	2-4%	0	0	0.027	0.037	0.014	0.002	0.001	0	0	0	0.08
	4-6%	0	0	0	0	0	0.004	0.002	0	0	0	0.01
**Better**	>6%	0	0	0	0	0	0	0	0	0	0	0.00
Prop better		0.49	0.50	0.52	0.54	0.53	0.53	0.57	0.00	0.00	0.00	**0.51**
Freq		0.418	0.266	0.164	0.092	0.036	0.015	0.007	0.000	0.000	0.000	
Cumulit Freq		0.418	0.684	0.848	0.94	0.976	0.991	0.998	0.998	0.998	0.998	

10

Eight linking videos, 100% Examiner Participation

Size of score adjustment

**Change in score accuracy**		0-1%	1-2%	2-3%	3-4%	4-5%	5-6%	6-7%	7-8%	8-9%	>9%	
**Worse**	>6%	0	0	0	0	0	0	0	0	0	0	0.00
	4-6%	0	0	0	0	0	0.001	0	0	0	0	0.00
	2-4%	0	0	0.02	0.025	0.008	0.001	0	0	0	0	0.05
	0-2%	0.221	0.123	0.047	0.011	0.004	0.001	0	0	0	0	0.41
	0-2%	0.227	0.145	0.058	0.012	0.005	0.001	0	0	0	0	0.45
	2-4%	0	0	0.026	0.036	0.014	0.001	0	0	0	0	0.08
	4-6%	0	0	0	0	0	0.003	0.001	0	0	0	0.00
**Better**	>6%	0	0	0	0	0	0	0	0	0	0	0.00
Prop better		0.51	0.54	0.56	0.57	0.61	0.63	1.00	0.00	0.00	0.00	**0.53**
Freq		0.448	0.268	0.151	0.084	0.031	0.008	0.001	0.000	0.000	0.000	
Cumulit Freq		0.448	0.716	0.867	0.951	0.982	0.990	0.991	0.991	0.991	0.991	

Study 2: Proportions of students receiving each category of score adjustment / change in accuracy

Zero percent baseline difference, 6 stations

Size of score adjustment

**Change in score accuracy**		0-1%	1-2%	2-3%	3-4%	4-5%	5-6%	6-7%	7-8%	8-9%	>9%	
**Worse**	>6%	0	0	0	0	0	0	0	0.002	0.002	0.003	0.01
	4-6%	0	0	0	0	0	0.022	0.012	0.004	0.001	0.001	0.04
	2-4%	0	0	0.034	0.053	0.033	0.006	0.003	0.002	0.001	0.001	0.13
	0-2%	0.127	0.111	0.059	0.012	0.008	0.006	0.004	0.002	0.001	0.001	0.33
	0-2%	0.127	0.109	0.054	0.012	0.007	0.006	0.004	0.002	0.001	0.001	0.32
	2-4%	0	0	0.031	0.05	0.033	0.005	0.003	0.002	0.001	0.001	0.13
	4-6%	0	0	0	0	0	0.018	0.011	0.005	0.001	0.001	0.04
**Better**	>6%	0	0	0	0	0	0	0	0.002	0.002	0.001	0.01
Prop better		0.50	0.50	0.48	0.49	0.49	0.46	0.49	0.52	0.50	0.40	**0.49**
Freq		0.254	0.22	0.178	0.127	0.081	0.063	0.037	0.021	0.01	0.01	
Cumulit Freq		0.254	0.474	0.652	0.779	0.86	0.923	0.96	0.981	0.991	1.001	

Zero percent baseline difference, 12 stations

Size of score adjustment

**Change in score accuracy**		0-1%	1-2%	2-3%	3-4%	4-5%	5-6%	6-7%	7-8%	8-9%	>9%	
**Worse**	>6%	0	0	0	0	0	0	0	0	0.001	0	0.00
	4-6%	0	0	0	0	0	0.008	0.003	0.001	0	0	0.01
	2-4%	0	0	0.031	0.039	0.025	0.004	0.001	0.001	0	0	0.10
	0-2%	0.179	0.132	0.058	0.014	0.009	0.004	0.001	0	0	0	0.40
	0-2%	0.174	0.125	0.055	0.015	0.009	0.004	0.001	0	0	0	0.38
	2-4%	0	0	0.027	0.038	0.022	0.004	0.001	0	0	0	0.09
	4-6%	0	0	0	0	0	0.006	0.002	0.001	0	0	0.01
**Better**	>6%	0	0	0	0	0	0	0	0	0	0	0.00
Prop better		0.49	0.49	0.48	0.50	0.48	0.47	0.44	0.33	0.00	######	**0.49**
Freq		0.353	0.257	0.171	0.106	0.065	0.03	0.009	0.003	0.001	0	
Cumulit Freq		0.353	0.61	0.781	0.887	0.952	0.982	0.991	0.994	0.995	0.995	

Zero percent baseline difference, 18 stations

Size of score adjustment

**Change in score accuracy**		0-1%	1-2%	2-3%	3-4%	4-5%	5-6%	6-7%	7-8%	8-9%	>9%	
**Worse**	>6%	0	0	0	0	0	0	0	0.004	0.006	0.007	0.02
	4-6%	0	0	0	0	0	0.028	0.019	0.008	0.003	0.002	0.06
	2-4%	0	0	0.038	0.07	0.055	0.012	0.006	0.004	0.002	0.002	0.19
	0-2%	0.119	0.119	0.068	0.022	0.016	0.01	0.006	0.003	0.002	0.001	0.37
	0-2%	0.106	0.089	0.048	0.018	0.012	0.008	0.005	0.002	0.001	0.001	0.29
	2-4%	0	0	0.018	0.026	0.017	0.005	0.003	0.001	0.001	0.001	0.07
	4-6%	0	0	0	0	0	0.005	0.002	0.001	0	0	0.01
**Better**	>6%	0	0	0	0	0	0	0	0	0	0	0.00
Prop better		0.47	0.43	0.38	0.32	0.29	0.26	0.24	0.17	0.13	0.14	**0.37**
Freq		0.225	0.208	0.172	0.136	0.1	0.068	0.041	0.023	0.015	0.014	
Cumulit Freq		0.225	0.433	0.605	0.741	0.841	0.909	0.95	0.973	0.988	1.002	

Five percent baseline difference, 6 stations

Size of score adjustment

**Change in score accuracy**		0-1%	1-2%	2-3%	3-4%	4-5%	5-6%	6-7%	7-8%	8-9%	>9%	
**Worse**	>6%	0	0	0	0	0	0	0	0.003	0.004	0.005	0.01
	4-6%	0	0	0	0	0	0.02	0.012	0.005	0.002	0.002	0.04
	2-4%	0	0	0.027	0.045	0.036	0.007	0.004	0.003	0.002	0.003	0.13
	0-2%	0.107	0.086	0.04	0.012	0.011	0.008	0.005	0.003	0.002	0.002	0.28
	0-2%	0.108	0.093	0.046	0.011	0.011	0.009	0.005	0.004	0.002	0.004	0.29
	2-4%	0	0	0.03	0.059	0.052	0.008	0.005	0.003	0.002	0.003	0.16
	4-6%	0	0	0	0	0	0.032	0.02	0.008	0.002	0.003	0.07
**Better**	>6%	0	0	0	0	0	0	0	0.005	0.007	0.01	0.02
Prop better		0.50	0.52	0.53	0.55	0.57	0.58	0.59	0.59	0.57	0.63	**0.54**
Freq		0.215	0.179	0.143	0.127	0.11	0.084	0.051	0.034	0.023	0.032	
Cumulit Freq		0.215	0.394	0.537	0.664	0.774	0.858	0.909	0.943	0.966	0.998	

Five percent baseline difference, 12 stations

Size of score adjustment

**Change in score accuracy**		0-1%	1-2%	2-3%	3-4%	4-5%	5-6%	6-7%	7-8%	8-9%	>9%	
**Worse**	>6%	0	0	0	0	0	0	0	0.001	0.001	0.001	0.00
	4-6%	0	0	0	0	0	0.012	0.006	0.002	0.001	0.001	0.02
	2-4%	0	0	0.022	0.04	0.03	0.007	0.004	0.002	0.001	0.001	0.11
	0-2%	0.104	0.079	0.045	0.02	0.014	0.008	0.005	0.003	0.001	0.001	0.28
	0-2%	0.11	0.109	0.063	0.021	0.015	0.01	0.006	0.003	0.002	0.001	0.34
	2-4%	0	0	0.041	0.074	0.055	0.01	0.006	0.003	0.002	0.001	0.19
	4-6%	0	0	0	0	0	0.024	0.016	0.006	0.001	0.001	0.05
**Better**	>6%	0	0	0	0	0	0	0	0.002	0.003	0.002	0.01
Prop better		0.51	0.58	0.61	0.61	0.61	0.62	0.65	0.64	0.67	0.56	**0.59**
Freq		0.214	0.188	0.171	0.155	0.114	0.071	0.043	0.022	0.012	0.009	
Cumulit Freq		0.214	0.402	0.573	0.728	0.842	0.913	0.956	0.978	0.99	0.999	

Five percent baseline difference, 18 stations

Size of score adjustment

**Change in score accuracy**		0-1%	1-2%	2-3%	3-4%	4-5%	5-6%	6-7%	7-8%	8-9%	>9%	
**Worse**	>6%	0	0	0	0	0	0	0	0.003	0.005	0.008	0.02
	4-6%	0	0	0	0	0	0.021	0.017	0.008	0.003	0.005	0.05
	2-4%	0	0	0.026	0.047	0.037	0.011	0.009	0.006	0.004	0.005	0.15
	0-2%	0.089	0.084	0.049	0.019	0.015	0.013	0.009	0.007	0.005	0.006	0.30
	0-2%	0.088	0.086	0.051	0.019	0.016	0.013	0.009	0.007	0.004	0.005	0.30
	2-4%	0	0	0.027	0.049	0.039	0.011	0.008	0.005	0.003	0.003	0.15
	4-6%	0	0	0	0	0	0.018	0.012	0.006	0.003	0.002	0.04
**Better**	>6%	0	0	0	0	0	0	0	0.001	0.002	0.002	0.01
Prop better		0.50	0.51	0.51	0.51	0.51	0.48	0.45	0.44	0.41	0.33	**0.49**
Freq		0.177	0.17	0.153	0.134	0.107	0.087	0.064	0.043	0.029	0.036	
Cumulit Freq		0.177	0.347	0.5	0.634	0.741	0.828	0.892	0.935	0.964	1	

Ten percent baseline difference, 6 stations

Change in score accuracy		Size of score adjustment 0-1%	1-2%	2-3%	3-4%	4-5%	5-6%	6-7%	7-8%	8-9%	>9%	
**Worse**	>6%	0	0	0	0	0	0	0	0.003	0.006	0.011	0.02
	4-6%	0	0	0	0	0	0.017	0.015	0.009	0.003	0.006	0.05
	2-4%	0	0	0.014	0.027	0.026	0.007	0.007	0.006	0.004	0.008	0.10
	0-2%	0.046	0.04	0.023	0.008	0.009	0.009	0.008	0.007	0.005	0.01	0.17
	0-2%	0.055	0.064	0.038	0.011	0.01	0.01	0.01	0.009	0.006	0.011	0.22
	2-4%	0	0	0.031	0.061	0.066	0.009	0.01	0.009	0.006	0.012	0.20
	4-6%	0	0	0	0	0	0.053	0.052	0.028	0.006	0.011	0.15
**Better**	>6%	0	0	0	0	0	0	0	0.015	0.025	0.048	0.09
Prop better		0.54	0.62	0.65	0.67	0.68	0.69	0.71	0.71	0.70	0.70	**0.67**
Freq		0.101	0.104	0.106	0.107	0.111	0.105	0.102	0.086	0.061	0.117	
Cumulit Freq		0.101	0.205	0.311	0.418	0.529	0.634	0.736	0.822	0.883	1	

Ten percent baseline difference, 12 stations

Change in score accuracy		Size of score adjustment 0-1%	1-2%	2-3%	3-4%	4-5%	5-6%	6-7%	7-8%	8-9%	>9%	
**Worse**	>6%	0	0	0	0	0	0	0	0.002	0.002	0.002	0.01
	4-6%	0	0	0	0	0	0.012	0.01	0.006	0.002	0.002	0.03
	2-4%	0	0	0.007	0.017	0.019	0.009	0.008	0.006	0.004	0.004	0.07
	0-2%	0.028	0.024	0.016	0.011	0.013	0.014	0.013	0.009	0.005	0.006	0.14
	0-2%	0.042	0.057	0.04	0.014	0.017	0.018	0.017	0.01	0.007	0.007	0.23
	2-4%	0	0	0.03	0.073	0.096	0.021	0.018	0.013	0.007	0.009	0.27
	4-6%	0	0	0	0	0	0.079	0.068	0.031	0.008	0.009	0.20
**Better**	>6%	0	0	0	0	0	0	0	0.015	0.021	0.024	0.06
Prop better		0.60	0.70	0.75	0.76	0.78	0.77	0.77	0.75	0.77	0.78	**0.75**
Freq		0.07	0.081	0.093	0.115	0.145	0.153	0.134	0.092	0.056	0.063	
Cumulit Freq		0.07	0.151	0.244	0.359	0.504	0.657	0.791	0.883	0.939	1.002	

Ten percent baseline difference, 18 stations

Change in score accuracy		Size of score adjustment 0-1%	1-2%	2-3%	3-4%	4-5%	5-6%	6-7%	7-8%	8-9%	>9%	
**Worse**	>6%	0	0	0	0	0	0	0	0.001	0.003	0.01	0.01
	4-6%	0	0	0	0	0	0.008	0.007	0.005	0.004	0.01	0.03
	2-4%	0	0	0.008	0.015	0.014	0.006	0.007	0.007	0.006	0.015	0.08
	0-2%	0.043	0.034	0.019	0.01	0.01	0.011	0.01	0.01	0.009	0.019	0.18
	0-2%	0.051	0.065	0.041	0.012	0.014	0.014	0.014	0.012	0.011	0.023	0.26
	2-4%	0	0	0.031	0.068	0.072	0.017	0.016	0.014	0.011	0.019	0.25
	4-6%	0	0	0	0	0	0.052	0.044	0.025	0.01	0.017	0.15
**Better**	>6%	0	0	0	0	0	0	0	0.011	0.015	0.023	0.05
Prop better		0.54	0.66	0.73	0.76	0.78	0.77	0.76	0.73	0.68	0.60	**0.70**
Freq		0.094	0.099	0.099	0.105	0.11	0.108	0.098	0.085	0.069	0.136	
Cumulit Freq		0.094	0.193	0.292	0.397	0.507	0.615	0.713	0.798	0.867	1.003	

Twenty percent baseline difference, 6 stations

Size of score adjustment

**Change in score accuracy**		0-1%	1-2%	2-3%	3-4%	4-5%	5-6%	6-7%	7-8%	8-9%	>9%	
**Worse**	>6%	0	0	0	0	0	0	0	0.001	0.003	0.019	0.02
	4-6%	0	0	0	0	0	0.002	0.004	0.003	0.003	0.013	0.03
	2-4%	0	0	0.003	0.004	0.004	0.002	0.002	0.003	0.004	0.02	0.04
	0-2%	0.005	0.006	0.006	0.003	0.003	0.002	0.003	0.004	0.005	0.028	0.07
	0-2%	0.008	0.014	0.012	0.004	0.003	0.003	0.004	0.005	0.007	0.039	0.10
	2-4%	0	0	0.009	0.019	0.025	0.003	0.005	0.006	0.007	0.046	0.12
	4-6%	0	0	0	0	0	0.032	0.044	0.032	0.01	0.057	0.18
**Better**	>6%	0	0	0	0	0	0	0	0.026	0.06	0.365	0.45
Prop better		0.62	0.70	0.70	0.77	0.80	0.86	0.85	0.86	0.85	0.86	**0.85**
Freq		0.013	0.02	0.03	0.03	0.035	0.044	0.062	0.08	0.099	0.587	
Cumulit Freq		0.013	0.033	0.063	0.093	0.128	0.172	0.234	0.314	0.413	1	

Twenty percent baseline difference, 12 stations

Size of score adjustment

**Change in score accuracy**		0-1%	1-2%	2-3%	3-4%	4-5%	5-6%	6-7%	7-8%	8-9%	>9%	
**Worse**	>6%	0	0	0	0	0	0	0	0	0.001	0.004	0.01
	4-6%	0	0	0	0	0	0	0.001	0.001	0.001	0.006	0.01
	2-4%	0	0	0.001	0.001	0.001	0	0.001	0.001	0.002	0.012	0.02
	0-2%	0.001	0.005	0.005	0.003	0.001	0.001	0.002	0.003	0.003	0.022	0.05
	0-2%	0.003	0.014	0.013	0.004	0.002	0.002	0.003	0.004	0.007	0.038	0.09
	2-4%	0	0	0.007	0.01	0.012	0.003	0.004	0.007	0.01	0.059	0.11
	4-6%	0	0	0	0	0	0.022	0.037	0.032	0.013	0.076	0.18
**Better**	>6%	0	0	0	0	0	0	0	0.027	0.072	0.441	0.54
Prop better		0.75	0.74	0.77	0.78	0.88	0.96	0.92	0.93	0.94	0.93	**0.92**
Freq		0.004	0.019	0.026	0.018	0.016	0.028	0.048	0.075	0.109	0.658	
Cumulit Freq		0.004	0.023	0.049	0.067	0.083	0.111	0.159	0.234	0.343	1.001	

Twenty percent baseline difference, 18 stations

Size of score adjustment

**Change in score accuracy**		0-1%	1-2%	2-3%	3-4%	4-5%	5-6%	6-7%	7-8%	8-9%	>9%	
**Worse**	>6%	0	0	0	0	0	0	0	0	0	0.002	0.00
	4-6%	0	0	0	0	0	0	0	0	0	0.005	0.01
	2-4%	0	0	0.001	0.001	0.001	0.001	0.001	0.001	0.001	0.011	0.02
	0-2%	0.007	0.005	0.003	0.002	0.002	0.002	0.001	0.001	0.002	0.023	0.05
	0-2%	0.01	0.014	0.011	0.004	0.003	0.002	0.002	0.002	0.003	0.042	0.09
	2-4%	0	0	0.009	0.02	0.031	0.002	0.003	0.005	0.006	0.064	0.14
	4-6%	0	0	0	0	0	0.038	0.054	0.035	0.01	0.086	0.22
**Better**	>6%	0	0	0	0	0	0	0	0.031	0.068	0.37	0.47
Prop better		0.59	0.74	0.83	0.89	0.92	0.93	0.97	0.97	0.97	0.93	**0.93**
Freq		0.017	0.019	0.024	0.027	0.037	0.045	0.061	0.075	0.090	0.603	
Cumulit Freq		0.017	0.036	0.06	0.087	0.124	0.169	0.23	0.305	0.395	0.998	
